# Effect of Marginal Designs on Fracture Strength of High Translucency Monolithic Zirconia Crowns

**DOI:** 10.1155/2020/8875609

**Published:** 2020-08-03

**Authors:** Niwut Juntavee, Sasiprapa Kornrum

**Affiliations:** ^1^Department of Prosthodontics, Faculty of Dentistry, Khon Kaen University, Khon Kaen 40002, Thailand; ^2^Division of Biomaterials and Prosthodontics Research, Faculty of Dentistry, Khon Kaen University, Khon Kaen 40002, Thailand

## Abstract

**Introduction:**

Monolithic zirconia is able to achieve certain aesthetic, but its durability in resisting fracture has been questioned, as fractures often originate from margins of restoration. This study determined fracture resistance of highly translucent monolithic zirconia crowns with different margin designs in terms of marginal thickness and collar height.

**Materials and Methods:**

Zirconia blanks (Ceramill® Zolid HT^+^) were selected for the fabrication of zirconia crowns according to different designs, including varying margin thicknesses (light chamfer, C_L_; heavy chamfer, C_H_) and collar heights (no collar, N_C_; low collar, L_C_; high collar, H_C_), which resulted in C_L_N_C_, C_L_L_C_, C_L_H_C_, C_H_N_C_, C_H_L_C_, and C_H_H_C_ groups (15 crowns each). The crowns were seated on a metal die and loaded vertically through round end punch (*θ* = 10 mm), contacting with inclined planes of cusp in a testing machine with crosshead speed of 0.2 mm/min until fracture. Videos with a rate of 50 frames/second were used to record fracture. Fracture load (N) and durable period (s) were compared for significant differences using ANOVA and Bonferroni test (*α* *=* *0.05*).

**Results:**

The mean ± sd of fracture load (N) and durable time (s) were 3211 ± 778 and 212 ± 47 for C_L_N_C_; 3041 ± 1370 and 188 ± 53 for C_L_L_C_; 2913 ± 828 and 192 ± 27 for C_L_H_C_; 4226 ± 905 and 245 ± 35 for C_H_N_C_; 4486 ± 807 and 228 ± 29 for C_H_L_C_; and 4376 ± 1043 and 227 ± 37 for C_H_H_C_. This indicated that marginal thickness had a significant influence on load-bearing capacity and durable time (*p* < 0.05). No significant impact of collar height was shown, either on load-bearing capacity or durable time (*p* > 0.05). No interaction between two factors was presented (*p* > 0.05).

**Conclusions:**

Heavy chamfer margin provided stronger zirconia crown than light chamfer, but both were capable of withstanding fracture load higher than maximum masticatory force. Neither presence nor absence of collar indicated any impact on strength. Fabrication of zirconia crowns with either heavy or light chamfer margin and either presence or absence of collar, with the consideration of emergence profile, should be considered.

## 1. Introduction

Nowadays, all ceramic restorations have been widely used for fixed partial dentures because of their biocompatibility and aesthetics outcomes. Zirconium dioxide (zirconia, ZrO_2_) has been introduced for use in restorative dentistry as a zirconia-based ceramic veneering prosthesis. The zirconia frameworks were predominantly fabricated in the form of yttria-stabilized tetragonal zirconia polycrystalline (Y-TZP) [1, 2]. Naturally, zirconia has a high melting temperature and low thermal conductivity. It was found in three crystallographic structures: the monoclinic (m-), tetragonal (t-), and cubic (c-) phase. The m-phase is quite stable at normal room temperature; however, when the temperature is raised up to 1170°C, it transforms into the t-phase [2, 3]. Eventually, zirconia can change to the c-phase when the temperature is raised to 2370°C [3]. Upon adding 3% mol of yttrium to zirconia, it can suppress the t- ⟶ m-phase transformation, allowing for the generation of the t-phase at room temperature. During the cooling process, small amounts of yttria oxides can be fully or partially stabilized, forming the metastable t-phase by not being transformed into a stable m-phase zirconia. However, a reversible transformation of t- ⟶ m-phase could possibly occur, enabling a volumetric expansion of about 4–5% [4]. This results in compressive stresses that resist crack generation, as well as enhancing crack propagation resistance, and is named the “*transformation toughening*” process [5, 6].

Zirconia restorations have been gaining much popularity as a result of advances in the dental technology of restoration fabrication using computer-assisted design (CAD) and computer-assisted manufacturing (CAM) processes that were capable of producing precise restorations [7]. Dental zirconia can be milled according to two different CAD and CAM techniques [8]. The first technique is a soft machining procedure, which is capable of shaping pre-sintered blocks into a slightly large or green state framework, which is then sintered in a sintering furnace to the desired dimension [9]. The second technique is the hard machining technique, which utilizes a fully sintered block that is shaped into the final dimensions of the restoration and does not need further sintering, and therefore, no shrinkage is presented [8]. However, zirconia is generally opaque in nature, thus the consideration of using zirconia restorations in the aesthetics zone has been very limited. Hence, it is necessary to veneer the zirconia framework with glassy porcelain to achieve positive aesthetics [2]. However, it was reported that the chipping and fracturing of this veneering ceramic is a major concern for long-term prognosis [10]. The current introduction of nonveneered zirconia, monolithic zirconia, into clinical practice aims to reduce the chipping and fracturing complications of zirconia ceramic restoration [11, 12]. The monolithic zirconia restoration is also fabricated by a soft milling method, and it can be used substantially in the posterior region.

Recently, the development of translucent zirconia has been introduced into dental practices, challenging the natural aesthetic appearance of zirconia, as it can be used solely in the aesthetic zone as monolithic high translucency zirconia restoration. This material demonstrates high strength levels, and it has been used increasingly in dental practices, especially for chair-side fabrications of zirconia restorations. However, it has been detected that the origin of failure for the zirconia crown fracture during clinical usage was reported to have been generated from the crown margin [13]. The margin is a crucial area of restoration that is closely adapted to the definite finishing area of the prepared abutment. The quality of the restoration margin is directly related to the tooth preparation technique and the process of restoration fabrication. Excellent clinical skills and techniques allow the clinician to digitally fabricate a well-contoured restoration with an acceptable marginal fit and a proper emergence profile. The emergence profile, termed as such by Stein and Kuwata in 1977, is demonstrated as a part of the axial contour of the tooth and restoration that emerges from the base of the gingival portion [14]. It was revealed that the proper shape of the emergence profile should be a straight line, which can be shown in a photographic analysis study of natural teeth [15]. An emerging unnatural contour, such as a convex or concave profile, may increase the accumulation of bacterial plaque and possibly interrupt the self-cleansing process around the marginal area of the restoration. The advocation of a straight emergence profile for restoration provides accessibility and efficiency in oral hygiene control, especially in a minute sulcular area of gingiva in a compromised periodontium situation [15, 16].

Regarding the fabrication of monolithic zirconia crowns, the marginal areas are areas of minimal thickness that frequently lead to the easy fracture of the crown [17]. The source of the failure originating in the margin of the zirconia restoration may relate to the margin design, as well as the thickness of the margins. Several investigations have been carried out with regard to the effect of the margin design on the load-bearing capacity of zirconia restoration in relation to occlusal thickness and wall thickness [13, 18–21]. Some investigations into the fracture resistance of all ceramic restorations were carried out by applying an occlusal load, either longitudinally or obliquely, on anatomical crowns until fracture, indicating that the fracture was possibly influenced by the design of the margin in the restoration [22–24]. A fractography study is the most reliable method to examine fractured surfaces, so a fractographic pattern was used in order to identify the material's weak points. It frequently applied into ceramic in dentistry to analyze the failure of ceramic restoration [17, 25]. The identifications of the failures of ceramic restorations were regularly indicated in the patterns by a compressive curl, hackle, wake hackle, twist hackle, and arrest lines, which contributed towards distinguishing the crack propagation pattern and the origin of failure [17]. Scanning electron photomicrographs revealed that the crack generally originates from the cervical margin [26–28]. Therefore, the design of the margins in restorations has a significant influence on the fracture resistance of the ceramic restoration. This idea was described in the numerical finite element analysis, which compared the stress dissemination during chewing between maxillary second premolars restored using porcelain-fused crowns, metal crowns, and nonrestored teeth. This showed that the stresses were concentrated at the finishing line of the restored teeth and, in the restoration, at the ceramic–metal interface [29]. Some studies advise fabricating the margin of restoration in the form of a chamfer and adding a collar design, as this has been discovered to be a suitable design for long-lasting restorations, especially in the posterior region [29, 30]. It was reported that ceramic-veneered zirconia restorations with customized collars at the cervical area of the zirconia substructure indicated a higher fracture toughness than the zirconia core without a collar design [30]. As previously described, the effects of marginal design and restoration configuration on fracture characteristics, however, are still certainly unclear in terms of optimal strength in a high-translucency monolithic zirconia (HTMZ) restoration.

Therefore, the objective of this experimental study was to compare the fracture load and durable time of fracture for monolithic zirconia crowns with different geometric designs of margins upon compressive loading. The null hypothesis was that different marginal designs of HTMZ restorations had no significant effect on fracture load or durable time for fracture strength.

## 2. Materials and Methods

### 2.1. Metal Die Fabrication

**Figure 1:**
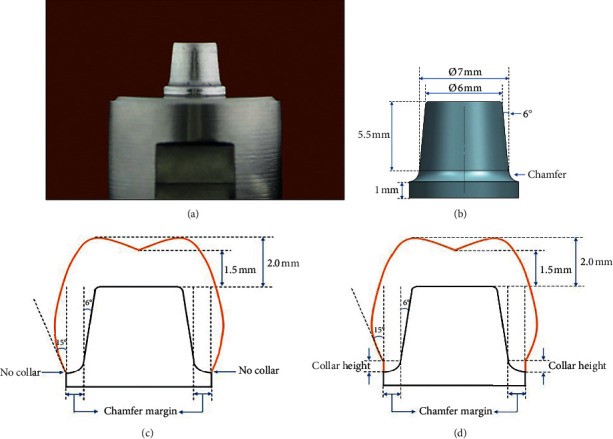
Stainless-steel dies were machined in cylindrical shape with chamfer margin (a) and possessed an axial surface height of 5.5 mm with 6-degree taper and round occlusal and cervical line angles of 6.0 mm and 7.0 mm in diameter, respectively (b). Zirconia crowns were fabricated on dies according to the thickness of chamfer margin, and either absence (c) or presence of collar (d), according to their respective dimensions.

Two sets of stainless-steel definitive dies were designed using the full-features of a three-dimension (3D) computer-assisted design application (Unigraphics NX2; Siemens PLM Software, TX, USA) and milled using a computer numerical control milling machine (Mikron VCE 750; Mikron AG Biel, Biel, Switzerland). Two stainless-steel dies were identically manufactured in a cylindrical shape to simulate molar abutment (Figure 1(a)); one with a light chamfer margin (C_L_) with a width of 0.8 mm and another with a heavy chamfer margin (C_H_) with a width of 1.2 mm. They possessed an axial surface height of 5.5 mm with a 6-degree taper, leading to a total convergence angle of 12 degrees. The abutment possessed round occlusal and cervical line angles with diameters of 6.0 mm and 7.0 mm, respectively (Figure 1(b)). The abutment was positioned on the cylindrical metal base and used as a master die for the fabrication of zirconia crowns.

### 2.2. Resin Die Fabrication

The metal dies were scanned using a digital scanner (Ceramill® map 400; Amann Girrbach AG, Koblach, Austria). The 3D printer (NextDent™ 5100; Amann Girrbach AG, Koblach, Austria) was used to fabricate the light polymerization resin dies. Six resin dies were fabricated with the monomer, which had a structure based on acrylic esters (NextDent Model 2.0; NextDent BV, Soesterberg, Netherlands) in order to replicate the definitive resin dies, which would be used in the process of the fabrication of zirconia crowns for each group.

### 2.3. Design of the Monolithic Zirconia Crowns

Ninety zirconia crowns were fabricated for six testing groups, based on the margin designs of the restorations that were related to the designs for the emergence profile present in Figures 1(c) and 1(d). The designs of the zirconia crowns varied according to the thickness of chamfer margin—the light chamfer (C_L_: 0.8 mm thickness) and the heavy chamfer (C_H_: 1.2 mm thickness)—as well as the height of cervical collar—no collar (N_C_: 0.0 mm collar height), low collar (L_C_: 0.5 mm collar height), and high collar (H_C_: 1.0 mm collar height)—which resulted in six groups of zirconia crowns: C_L_N_C_, C_H_N_C_, C_L_L_C_, C_H_L_C_, C_L_H_C_, and C_H_H_C_ groups as shown in Table 1 (with 15 crowns in each group).

### 2.4. Fabrication of the Monolithic Zirconia Crowns

Six resin dies for each group were digitally scanned using the digital scanner (Ceramill® map 400; Amann Girrbach AG, Koblach, Austria), and they were used to design the zirconia crowns with the computer-assisted designed (CAD) software (Ceramill® mind; Amann Girrbach AG, Koblach, Austria). The digitally designed crowns for each group were confirmed as having an identical anatomy, contour, and emergence profile using the computerized control milling in CAD-wax (Ceramill® wax; Amann Girrbach AG, Koblach, Austria). The digital files of each restoration were transferred to the CAM to be milled with the green stage zirconia blocks (Ceramill® Zolid HT^+^ White; Amann Girrbach AG, Koblach, Austria). The pre-sintered milled zirconia restoration was performed in an oversize dimension for each model, in order to compensate for the dimensional shrinkage of 25–30%. They were then sintered in a furnace (Ceramill® Therm 3; Amann Girrbach AG, Koblach, Austria), according to the manufacturer's recommended firing parameters, at a temperature of 1450°C for a 120-minute holding period, which concluded with a sintering process that took a total of 7.5 hours for each zirconia crown. After the sintering process, the sintered restorations were tried, adjusted, and finished on the resin die until completely seated.

### 2.5. Fracture Strength Test

**Figure 2:**
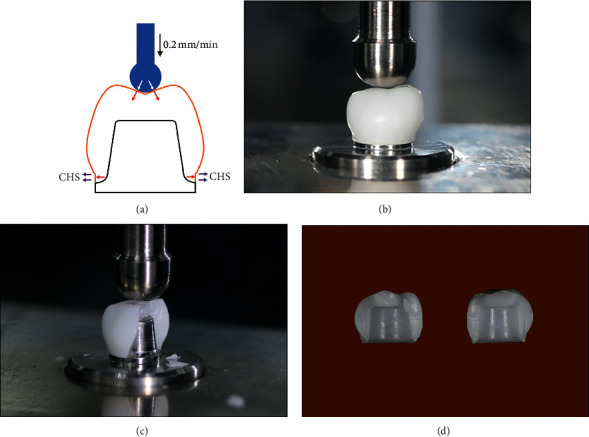
Zirconia crown was seated on metal die, loaded vertically from the occlusal surface in a universal testing machine with a round end hard steel punch at a crosshead speed of 0.2 mm/min to induce circumferential hoop stress (CHS) at the crown margin (a, b) until fracture (c). Fracture specimens were further examined microscopically for the analysis of fracture (d).

The fracture strength of each zirconia crown was determined on the master metal die. The zirconia crown was absolutely seated on its respective metal die without cementation. The load was vertically applied from the occlusal surface of the zirconia crown along the long axis of the tooth using a universal testing machine (UTM, Lloyd®, LR30/K, Leicester, England). This machine used a round-end (10 mm diameter) hard steel punch at a crosshead speed of 0.2 mm/min, in order for the circumferential hoop stress at the crown margin to develop, as presented in Figure 2(a). The fracture load and durable time that induced the ultimate cracks of each group were investigated in conjunction with the use of a video camera (Canon EOS 750D; Canon, Tokyo, Japan) at an optimal rate of 50 frames per second (s), until it reached its ultimate crack. The latter data were then viewed in relation to the load-displacement curve recorded by the UTM. Two mirrors (6 × 6 inches) were placed in an oblique position to visually maximize the observation of the crack around the zirconia crown. The video recordings were analyzed in slow motion to track the onset of the crack until the ultimate cracks occurred, as presented in Figures 2(b) and 2(c). An abrupt fall in the load-displacement curve was used to confirm the load (Newton, N) and time (second, s) of the failure for each specimen. The compressive load (N) at the point of failure and the durable period until reaching fracture (s) for each zirconia crown was recorded.

### 2.6. Fracture Surface Analysis

Upon the ultimate crack, the fractured specimens were retrieved and investigated with a stereomicroscope (Nikon Measurescope 20, Tokyo, Japan) at 50–100 magnification, using different light sources to identify the origins of the fracture, which were then further investigated for fracture characteristics under a scanning electron microscope (Hitachi S-300N, Osaka, Japan). The fractured specimens (Figure 2(d)) were mounted on a metal stub and were coated with gold-palladium (TK8842 Gold Target; Emitech, Ashford, UK) using a sputter-coating machine (K-500X; Emitech, Ashford, UK). The crack initiation defect of each specimen was located, and the patterns of the fracture were observed.

### 2.7. Statistical Analysis

The mean and standard deviation (sd) of the compressive load (N) at the point of failure and the time until fracture (s) of each group of zirconia crowns were calculated and then further analyzed using a two-way analysis of variance (2-way ANOVA). This was calculated in conjunction with a post hoc Bonferroni multiple comparison, which used statistical software (SPSS version 22; SPSS, Chicago, IL, USA) to determine the significant differences between the fracture strength and the duration of the fracture among all groups. The Weibull analysis was then performed to determine the strength of reliability using Weibull^++^® statistics (ReliaSoft, Tucson, AZ, USA), in order to estimate the characteristic strength (*σ*_o_) and the Weibull modulus (m).

## 3. Results

**Figure 3:**
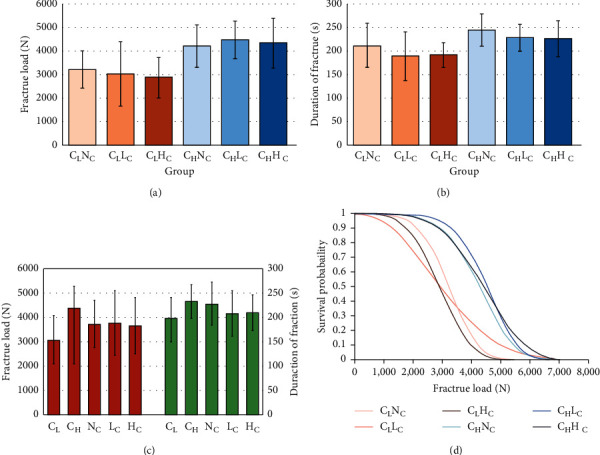
Fracture load (a, c), duration of fracture (b, c) of zirconia crowns, fabricated according to different thicknesses of chamfer margin (light chamfer, C_L_; heavy chamfer, C_H_) and collar heights (no collar, N_C_; low collar, L_C_; high collar, H_C_), and Weibull analysis for survival probability at different fracture loads for each group (d).

The mean, sd, 95% confidence interval, and Weibull modulus of fracture strength (N) for each group are illustrated in Table 1 and Figure 3(a). The highest compressive load at the point of fracture was demonstrated in the group C_H_L_C_, followed by C_H_H_C_, C_H_N_C_, C_L_N_C_, C_L_L_C_, and C_L_H_C_. The mean ± sd values of the compressive load at the point of fracture (N) of the zirconia crowns with C_L_ and C_H_ designs were 3055 ± 1012 and 4362 ± 909, respectively. The mean ± sd values of the compressive load at the point of fracture (N) of zirconia crowns with N_C_, L_C_, and H_C_ designs were 3718 ± 977, 3764 ± 1327, and 3644 ± 1187, respectively, as shown in Figure 3(c). A two-way ANOVA indicated a statistically significant difference in compressive load at the point of fracture due to a difference in marginal thickness of the chamfer margin (*p* < 0.05). However, the varied marginal collar design and the interaction between the marginal thickness and marginal collar designs did not indicate a significant influence on the compressive load at the point of failure of the zirconia crown, as presented in Table 2. The independent sample *T*-tests demonstrated that the different marginal thickness showed significant differences (*p* < 0.05) in the compressive load at the point of fracture, as presented in Table 3. Post hoc Bonferroni multiple comparisons demonstrated that there was not a significant difference (*p* > 0.05) in the value of the compressive load at the point of fracture in different collar designs, as presented in Table 3. Furthermore, a combination of different marginal thicknesses and different marginal collar designs produced significant differences in the compressive load at the point of fracture (*p* < 0.05), only between C_L_N_C_-C_H_L_C_, C_L_N_C_-C_H_H_C_, C_L_L_C_-C_H_N_C_, C_L_L_C_-C_H_L_C_, C_L_L_C_-C_H_H_C_, C_L_H_C_-C_H_N_C_, C_L_H_C_-C_H_L_C_, and C_L_H_C_-C_H_H_C_, as presented in Table 3. The Weibull analysis of the values of the compressive load at the point of fracture illustrated the “*m*” from highest to lowest, as C_H_N_C_ (5.04); C_L_N_C_ (4.59); C_L_H_C_ (3.81); C_H_H_C_ (3.72); C_H_L_C_ (3.40); and C_L_L_C_ (2.31), which indicated the relative survival probability of fracture in each group, as shown in Table 1 and Figure 3(d).

The mean ± sd values of durable time until fracture (s) were 212 ± 47 for C_L_N_C_; 188 ± 53 for C_L_L_C_; 192 ± 27 for C_L_H_C_; 245 ± 35 for C_H_N_C_; 228 ± 29 for C_H_L_C_; and 227 ± 37 for C_H_H_C_, as presented in Table 1 and Figure 3(b). The maximum time until fracture was demonstrated in the group C_H_N_C_, followed by C_H_L_C_, C_H_H_C_, C_L_N_C_, C_L_H_C_, and C_L_L_C_, respectively. The mean ± sd values of the time until fracture (s) with C_L_ and C_H_ designs were 197 ± 44 and 233 ± 34, respectively. The mean ± sd values of the time until fracture (s) with N_C_, L_C_, and H_C_ designs were 228 ± 44, 208 ± 47, and 210 ± 37, respectively, as shown in Figure 3(c). A two-way ANOVA indicated a statistically significant difference (s) due to a difference in marginal thickness of the chamfer margin (*p* < 0.05). However, the varied collar design and the interaction between marginal thicknesses and marginal collar designs did not significantly influence the amount of time until the fracture of the zirconia crowns (*p* > 0.05), as shown in Table 2. An independent sample *T*-test demonstrated that the different marginal thicknesses showed significant influence on the amount of time until the fracture (s) of the zirconia crowns (*p* < 0.05), as presented in Table 3 and Figure 3(c). Post hoc Bonferroni multiple comparisons demonstrated that the time until the fracture of the crowns related to the different collar designs did not indicate a significant difference (*p* > 0.05), as presented in Table 3 and Figure 3(c). In addition, a combination effect of different marginal thicknesses and different collar designs produced a significant impact on the time until fracture (*p* < 0.05), only between the C_L_L_C_ group and the C_H_N_C_ group, as presented in Table 3 and Figure 3(b).

**Figure 4:**
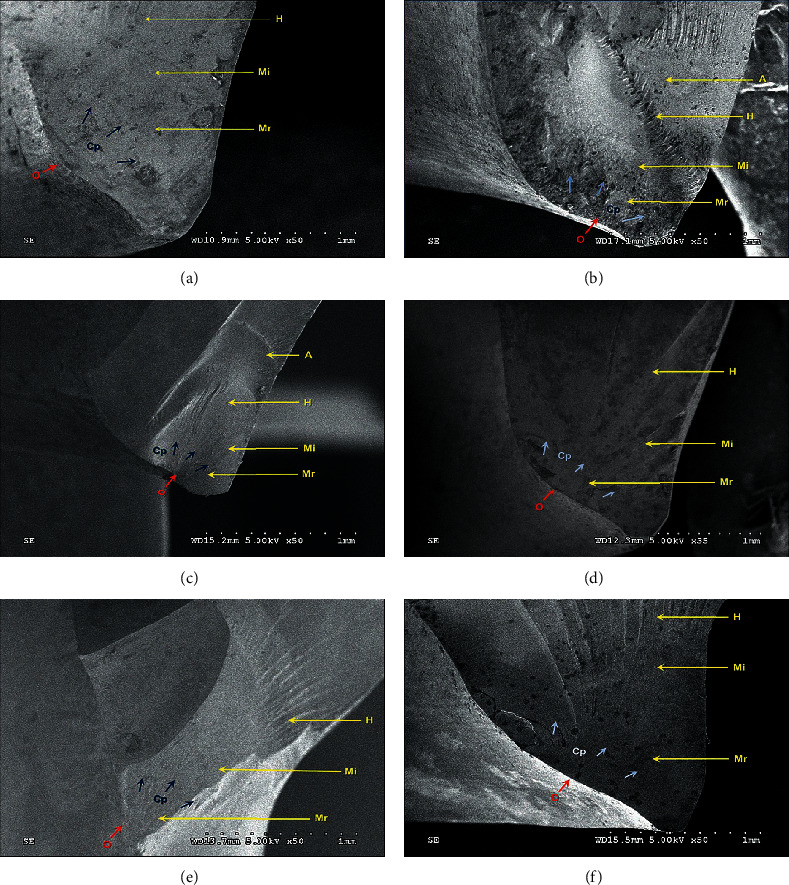
SEM photomicrographs of fracture surfaces at X50 magnification (a–f) indicated fracture origin (O), mirror zone (Mr), mist zone (Mi), hackle zone (H), and arrest line (a–f) of zirconia crown fabricated with light chamfer (a, c, e) and heavy chamfer (b, d, f), in accordance with no collar (a, b), low collar (c, d), and high collar (e, f).

The fractographic analysis of the fracture surface, according to the SEM photomicrographs, indicated patterns of fracture clues in the monolithic zirconia crowns of each group, showing an area of mist, arrested lines, and hackle features, as presented in Figures 4(a)–4(f). All specimens clearly indicated the fact that the fracture originated from the margin area and was occlusally propagated along the axial surface of the zirconia crown as well as divergently deviated through the external surface of the crown until failure. The divergent deviation of the crack patterns from the origin of the fracture through the external surface of the crown was seen more rapidly in the zirconia crown with a light chamfer margin (Figures 4(a), 4(c) and 4(e), rather than the heavy chamfer margin (Figures 4(b), 4(d) and 4(f)).

## 4. Discussion

This study has indicated that the thickness of the margin design significantly affects the load-bearing capacity and durable time until fracture of HTMZ crowns. However, the design of collar did not have any influence on the load-bearing capacity and time until fracture of HTMZ crowns. Thus, the null hypotheses were rejected for the thickness of the chamfer margin but accepted for the collar design. The impact of the C_H_-margin over the C_L_-margin on the marginal endurance of the crown to resist fracture was possibly related to the marginal thickness, which enhanced the fracture resistance of the monolithic zirconia crowns. This seems to show a preference towards the heavy chamfer margin over the light chamfer margin for monolithic Y-TZP restoration. This finding is in accordance with other previous studies [18–21]. Although the increased thickness provides a stronger crown, the strength of fracture in the zirconia crowns in every group of this study showed a higher level of load-bearing capacity than the maximum mastication force of humans (850 N) [20]. Therefore, the light chamfer margin design would still be clinically acceptable for HTMZ crowns and it can be selectively used in appropriate situations, such as abutment with periodontal compromised situation. The light chamfer margin design also benefits gingival health, as it decreases the overcontouring of the crown margin and decreases the amount of tooth preparation required for the abutment tooth, which eventually effects periodontal health [18]. This study was supported by other studies that suggested that the tooth abutment should be prepared on the axial wall with a slight chamfer margin design, with 0.5 mm being a suitable margin depth for monolithic Y-TZP crowns [21]. In this study, the design of the collar for the margin of the zirconia crown did not have any significant effect on the load-bearing capacity of HTMZ in resisting fracture. The study strongly indicated that the presence of the collar did not jeopardize the strength of HTMZ crowns at all, in comparison to designs with no collar. On the contrary, the presence of a collar design in this study tends to provide more biological advantages for periodontium, over those with no collar design, in terms of the emergence profile of the zirconia restoration being correctly fabricated in a straight profile, replicating natural teeth, thus providing better biological compatibility of zirconia to gingival tissue, as supported by other studies [16, 31].

In terms of the duration of time until fracture, the study indicated that the heavy chamfer margin design (C_H_-group) was capable of surviving for a longer period before fracturing upon bearing load than the light chamfer margin design (C_L_-group). Thus, the difference in marginal design significantly affected the duration that the crown is able to resist fracture. In this study, the absence of a collar design on the HTMZ crown did not exhibit any ability to resist fracture for a longer duration than those with a collar, regardless of whether the collar was high or low. The study clearly indicated that the presence of a collar design in HTMZ crowns was absolutely possible without any reduction to the period that the crown can withstand a fracture. Thus, the absence of a collar design tends to induce more biological disadvantages for periodontium than the presence of collar design. The absence of a collar tends to result in the overcontouring of the crown at the margin, and it does not fabricate the correct emergence profile for the HTMZ crown to simulate the natural contouring of the teeth [16].

The ultimate load-bearing capacity of the HTMZ crowns has been quested for clinician for the purpose of using these crowns in practice, as the disadvantage of using all ceramic restorations is that fractures often occur, the majority of which start in the margin. The most regularly used margin for ceramic restorations is a chamfer margin with either a heavy or light chamfer. Thus, the study was designed to investigate the effect of margin design on the load-bearing capacity of the HTMZ in resisting fractures, and the period that the fracture load could be withstood. The heavy chamfer is generally used for conventional ceramic restorations of normal periodontal health, and the latter is often used in cases of a compromised periodontium. Yet, both designs need to be beneficial for periodontal heath in terms of the emergence profile. This study has clearly been created in order to answer these specific issues. By designing the investigation of load resistance to fracture, the zirconia crowns need to be supported by metal dies in order to be ensured of having a strong supporter to derive for ultimate fracture load for fracturing crown without any destroying on the supporter. All HTMZ crowns were seated on the metal die supported during testing without cementation owing to reach the objective of inducing the circumferential hoop stress on the margin to induce crack initiated from the margin and elimination of any confounding factors from the chemical and mechanical properties of cement that might affected fracture resistance [23, 32]. This study was designed using the spherical indenter of *θ* = 10 mm, so the contact point of the spherical indenter was rightly placed on the incline planes of the buccal and lingual cusps of the crown, thereby simulating the contact points of natural teeth during mastication. Other studies may recommend the use of a spherical indenter with *θ* ≥ 10 mm to create a broader contact area [28]; however, this study aimed to have the correct tripodization pattern of occlusion on the incline plane to induce compressive stress on the cusps and transfer the stress to the marginal area of the crown, thus inducing hoop stress at the margin without prematurely inducing cusp fracturing. This strongly indicates that the goal of this investigation has been achieved. The evidence of the testing was confirmed using a fractographic fracture analysis of the surface of the zirconia crown, indicating that the failure originated in the crown margin, with the crack patterns penetrating vertically and diagonally through the occlusal and external surfaces of the crown, similar to clinical fail zirconia restoration, and supported by other studies [27, 30]. In these findings, it was interesting that the crack propagation pattern in the light chamfer margin design was demonstrated diagonally on the external surface of the crown faster than that in the heavy chamfer margin design, which rather supports the evidence of a stronger load-bearing capacity and period of resistance to fracture in C_H_, compared with C_L_. The capacity of load withstanding of HTMZ crown for each group was confirmed for reliability as the Weibull modulus (*m*) was within the range of customary dental ceramic materials used in dentistry [19]. As such, this in vitro study could not simulate the human oral condition, which may present other factors that could affect the mechanical properties of monolithic zirconia restoration.

Crown design must be resistant enough to fracture to endure mastication and ensure positive periodontal health. This study has clearly indicated that chamfer margin design, both light and heavy, fulfilled the requirement for the load-bearing capacity of HTMZ restorations to be satisfactorily used in clinical practice. The absence of a collar design did not enhance the fracture strength of the HTMZ crown, in comparison to the presence of collar, regardless of whether the collar was high or low. Thus, this study recommends that the collar design should be advocated for HTMZ crowns, so as to ensure good periodontal health and the natural simulation of tooth contours in an appropriate emergence profile, especially in clinical situations when using a zirconia restoration on a patient with a compromised periodontal health. Accordingly, only in the limited clinical situations in which the restoration is being carried out on a patient with a healthy periodontal situation should the absence of a collar be acceptable; however, it was suggested that the emergence profile of the restoration should always be considered, in order not to affect a patient's periodontal health.

## 5. Conclusions

The study has indicated that the design of heavy chamfer margins provided a stronger and more durable zirconia crown than light chamfer margin. Nevertheless, both heavy and light chamfer margins were capable of withstanding a fracture load that is higher than the maximum masticatory force of humans. The design of a zirconia crown without a collar did not demonstrate a better load-bearing capacity or level of endurance, in comparison with those with a collar, regardless of whether it was a high or low collar. However, the presence of a collar seems to provide a favourable emergence profile, in comparison with the absence of collar, which suggests that the collar design should be routinely advocated for in HTMZ crowns. Using a zirconia crown with no collar should be limited to cases of healthy periodontal health.

## Figures and Tables

**Table 1:** Mean, standard deviation (sd), 95% confidential interval (CI), characteristic strength (*σ*_o_) of fracture strength (Newton, N), Weibull modulus (m), and duration of fracture (second, s) of high translucency monolithic zirconia upon different margin designs, including margin thickness (light chamfer, C_L_; heavy chamfer, C_H_) and collar height (no collar, N_C_; low collar, L_C_; high collar, H_C_).

Group	Fracture load (N)				*m*	Duration of fracture (s)		
	Mean ± sd	95% CI		*σ *_O_		Mean ± sd	95% CI	
		LB	UB				LB	UB
C_L_N_C_	3211 ± 778	2780	3641	3,508.73	4.61	212 ± 47	186	238
C_L_L_C_	3041 ± 1370	2282	3800	3,473.30	2.28	188 ± 53	159	218
C_L_H_C_	2913 ± 828	2454	3371	3,231.11	3.83	192 ± 27	177	207
C_H_N_C_	4226 ± 905	3724	4727	4,613.57	5.04	245 ± 35	225	264
C_H_L_C_	4486 ± 807	4039	4933	4,830.64	5.93	228 ± 29	211	244
C_H_H_C_	4376 ± 1043	3798	4953	4,801.51	4.40	227 ± 37	207	248

LB = lower bound; UB = upper bound.

**Table 2:** Two-way analysis of variance (ANOVA) of fracture load and for duration of fracture for high translucency monolithic zirconia crowns upon different margin designs, including margin thickness and collar height.

Source	SS	d*f*	MS	*F* test	*p* value
*ANOVA of fracture load*					
Corrected model	39635561.786^a^	5	7927112.357	8.303	0.001
Intercept	1237851270.774	1	1237851270.774	1296.593	0.001
Margin thickness	38456289.734	1	38456289.734	40.281	0.001
Collar height	217577.100	2	108788.550	0.114	0.892
Margin thickness *∗* collar height	961694.952	2	480847.476	0.504	0.606
Error	80194384.220	84	954695.050		
Total	1357681216.780	90			

*ANOVA for duration of fracture*					
Corrected model	36594.033^a^	5	7318.807	4.767	0.001
Intercept	4174420.226	1	4174420.226	2718.983	0.001
Margin thickness	28768.491	1	28768.491	18.738	0.001
Collar height	7656.821	2	3828.410	2.494	0.089
Margin thickness *∗* collar height	168.721	2	84.361	0.055	0.947
Error	128964.127	84	1535.287		
Total	4339978.386	90			

SS = sum of squares; d*f* = degree of freedom; MS = mean square; *F* = *F*-ratio; *p* = *p* value.

**Table 3:** Post hoc Bonferroni multiple comparisons of flexural strength (A, B, C) and duration of fracture (D, E, F) for high translucency monolithic zirconia crowns upon different margin designs, including margin thickness (light chamfer, C_L_; heavy chamfer, C_H_) and collar height (no collar, N_C_; low collar, L_C_; high collar, H_C_).

Post hoc multiple comparisons of flexural strength						
(A) As a function of margin thickness			(B) As a function of collar designs			
Thickness	C_L_	C_H_	Design	N_C_	L_C_	H_C_
C_L_	1	0.001	N_C_	1	1	1
C_H_		1	L_C_		1	1
			H_C_			1
(C) As a function of margin thickness and collar height						
Group	C_L_N_C_	C_L_L_C_	C_L_H_C_	C_H_N_C_	C_H_L_C_	C_H_H_C_
C_L_N_C_	1	1	1	0.084	0.009	0.024
C_L_L_C_		1	1	0.020	0.002	0.005
C_L_H_C_			1	0.006	0.001	0.001
C_H_N_C_				1	1	1
C_H_L_C_					1	1
C_H_H_C_						1
Post hoc multiple comparisons for duration of fracture						
(D) As a function of margin thickness			(E) As a function of collar designs			
Thickness	C_L_	C_H_	Design	N_C_	L_C_	H_C_
CL	1	0.001	N_C_	1	0.204	0.279
CH		1	L_C_		1	1
			H_C_			1
(F) As a function of margin thickness and collar height						
Group	C_L_N_C_	C_L_L_C_	C_L_H_C_	C_H_N_C_	C_H_L_C_	C_H_H_C_
C_L_N_C_	1	1	1	0.377	1	1
C_L_L_C_		1	1	0.003	0.110	0.118
C_L_H_C_			1	0.006	0.219	0.233
C_H_N_C_				1	1	1
C_H_L_C_					1	1
C_H_H_C_						1

## Data Availability

The data used to support the findings of this study are included within the article.
